# Planning for an aging nursing workforce: data-driven implications for health policy and service sustainability in Italy

**DOI:** 10.3389/frhs.2026.1778755

**Published:** 2026-03-27

**Authors:** Luca Fimmanò, Marco Damonte Prioli, Fabrizio Figallo, Giovanni Orengo, Antonio Uccelli, Michele Messmer

**Affiliations:** IRCCS Ospedale Policlinico San Martino, Genoa, Italy

**Keywords:** aging workforce, nursing demographics, occupational health, public healthcare systems, workforce planning

## Abstract

**Introduction:**

Nurses are a vital component of healthcare systems, directly influencing the quality and continuity of patient care. Globally, demographic shifts have led to a rising proportion of older nurses. In Italy, this trend presents challenges, given the rapidly aging population and ongoing workforce shortages. With a retirement age of 67, many older nurses remain in physically demanding roles despite medically documented work limitations, raising concerns about workforce sustainability, safety, and long-term planning.

**Methods:**

This study analyzed demographic trends among nurses at a major Italian public hospital. Data were extracted from the hospital's human resources system (IRIS WIN) for the period 2010–2024. A total of 2,184 nurses employed as of 31 December 2024 were stratified into four age groups (24–44, 45–54, 55–58, and 59–67 years) and mapped against clinical settings with varying levels of work intensity. Retirement eligibility was projected through 2033. Health-related absenteeism and medically documented work limitations were analyzed by age group and job intensity level.

**Results:**

Between 2010 and 2024, the proportion of nurses aged 24–44 declined by 36.6%, while those aged 55–58 and 59–67 increased by 222.3% and 1,914%, respectively. Projections indicate a further 91% increase in the oldest age group by 2033. In 2024, 66% of nurses aged 55–58 and 61% of those over 59 were working in high- or extremely high-intensity clinical areas. Older nurses (>55 years), representing 40% of the workforce, accounted for 54% of all health-related absenteeism, equivalent to 87 full-time nursing positions. The number of nurses with medically documented work limitations in the oldest age group is expected to double in the coming years.

**Discussion:**

This study highlights the urgent challenges of an aging nursing workforce which are not unique to Italy's NHS, but are a global issue. A growing proportion of older nurses, many with medically documented work limitations, are working in demanding clinical environments, trends already testing healthcare systems in many countries worldwide with a potential impact on patient safety, quality of care, and workforce resilience. Urgent investment in workforce planning, age-responsive role adaptation, and transitional pathways is essential to ensure sustainable, high-quality care delivery and to safeguard workforce health.

## 1 Introduction

Nurses are a vital component of healthcare delivery, directly impacting the quality and continuity of patient care. In recent decades, demographic changes have led to a rising proportion of older nurses globally (1). This trend presents challenges, as aging nurses may face specific health issues and workplace demands that affect their performance, well-being, and career longevity (2). The broader context includes significant demographic shifts across Italy and other countries, where aging populations and healthcare workforce shortages are creating systemic pressures (3).

Raising the retirement age to 67 in Italy is primarily aimed at ensuring the long-term financial stability of the pension system, facing an aging and shrinking population (4). In theory, within the national healthcare system, the measure should also aid in retaining experienced staff amid rising care demands and a declining labour pool.

However, extending nurses’ careers has complex implications due to the physical and psychological demands of the profession. It may affect health, job satisfaction, and performance (5, 6). Knowing how policy changes affect careers and the workplace is key to planning well and keeping healthcare services sustainable over time (7).

In Italy, where population aging is particularly noted, understanding the dynamics of an aging nursing workforce is critical, especially for large public hospitals employing an extensive nursing staff, responsible for maintaining high standards of care. This study examines the progression of this demographic in one hospital setting, the roles and departments where older nurses continue to work, and the health-related absenteeism and medically documented work limitations they experience.

This study has four aims: to analyse trends in the aging nurse population within a major Italian public hospital and project the future trajectory of this demographic; examine the distribution of older nurses across different hospital units; investigate the frequency of medically documented work limitations affecting attendance among aging nurses, and provide insights to support workforce planning and policy development for sustainable healthcare delivery that are adaptable to healthcare settings globally.

## 2 Methods

The San Martino Research Hospital in Genoa, a hospital within the national healthcare system (NHS), has approximately three million outpatient visits, 77,000 inpatient admissions and 80,000 emergency department visits yearly. Anonymized data on all nurses, full-time and part-time, were extracted from the hospital's human resources database, IRIS WIN, from 2010 through 2024. We conducted repeated cross-sectional analyses for a stable workforce of *N* = 2,184 nurses, where annual hires equaled exits, enabling observation of aging trends via yearly age distributions. Aging projections used a cohort-component model on the December 31, 2024 cohort, simulating each nurse's age trajectory under fixed N. Retirement eligibility was defined as age 67 (national pension age). Exits were offset by age-parameterized new hires matching 2010–2024 patterns to maintain headcount balance.

Percentages were calculated within each age group to show the proportion of nurses in that category. This method enables comparisons relative to each group's size and characteristics, avoiding distortions from aggregating across the total population, where narrower ranges (like late career) would be underrepresented due to unequal widths.

In order to differentiate the nature and intensity of the job demands, work settings were divided into 5 categories based on different levels of physical and cognitive effort required: administrative and out-patient clinic (low intensity), day hospital (medium intensity; refers to a hospital admission for testing or treatment, without requiring an overnight stay), in-patient ward (high intensity) and critical areas including intensive care and emergency (extremely high-intensity). Table 1 provides a description of each intensity level and examples of hospital departments categorized within each level. All medically documented work limitations were included and defined as workplace accommodations deemed necessary due to a health condition certified by an occupational health physician. These accommodations pertain to restrictions on manual patient lifting, night shift work, prolonged standing and exposure to allergenic or otherwise harmful agents. An occupational health information system is used to document and classify these limitations, which are reviewed and renewed during mandatory periodic assessments conducted by an occupational health physician. To project the number of medically documented work limitations among nurses in 2033 by age group, we used data from 2010 to 2024 and applied a cohort-component projection method, which extrapolates historical age-specific rates forward over time.

**Table 1:** Description of various intensity levels of nursing roles in hospital departments based on physical and cognitive effort required.

Intensity level	Description	Examples of Departments included under the description
Administration low	Predominantly administrative positions that require healthcare knowledge	Training & Education Clinical Governance Quality Assurance Public relations Risk Prevention Accreditation
Low	Out-patient setting for diagnosis and follow-up that requires minimal physical effort and moderate cognitive effort	All out-patient clinics
Moderate	Day Hospital: refers to a planned daytime admission used for multispecialty assessments, medical therapies (including some oncologic treatments), minor procedures, and pre- or post-operative care; requires moderate physical effort and high cognitive effort	Orthopedics Radiology Dermatology Oncology Neurology Gastroenterology Allergology
High	Hospital ward setting where patients are admitted for overnight stays; require high physical and cognitive effort	All inpatient hospital wards
Extremely high	Critical area with fast-paced environments; requires extremely high physical and cognitive effort	Intensive Care Unit Emergency Department Surgery Transplantation

Days of absenteeism were converted into full-time equivalents (FTE) assuming a total of 222 actual working days in a year. Both work limitations and health-related absenteeism were analyzed by age group and work setting. Analyses of health-related absenteeism were based on data from 2024, the most recent year for which complete records were available at the time of the study.

## 3 Results

### 3.1 Age

In 2024 there were 2,184 nurses on staff with a mean age of 50.49 years. Thirty-two percent of nurses were in the youngest age category (*n* = 699, 22–44 years), 28% (*n* = 614) were between 45 and 54 years of age, 21% (*n* = 448) between 55 and 58 years and 19% (*n* = 423) were 59–67 years.

Assessing the number of nurses in each age category between 2010 and 2024, nurses in the 24–44 years of age category decreased by 36.6%, from 1,102 to 699. The 45–54 years age category decreased by 19.5%, from 763 to 614. For the same period there were substantial increases in the more advanced age categories. In the 55–58 years category, the number of nurses increased from 139 to 448, representing a 222.3% increase. The number of nurses aged 59 to 67 years also increased substantially from 21 in 2010 to 423 in 2024, representing a 1,914% increase over the 14-year period.

**Figure 1:**
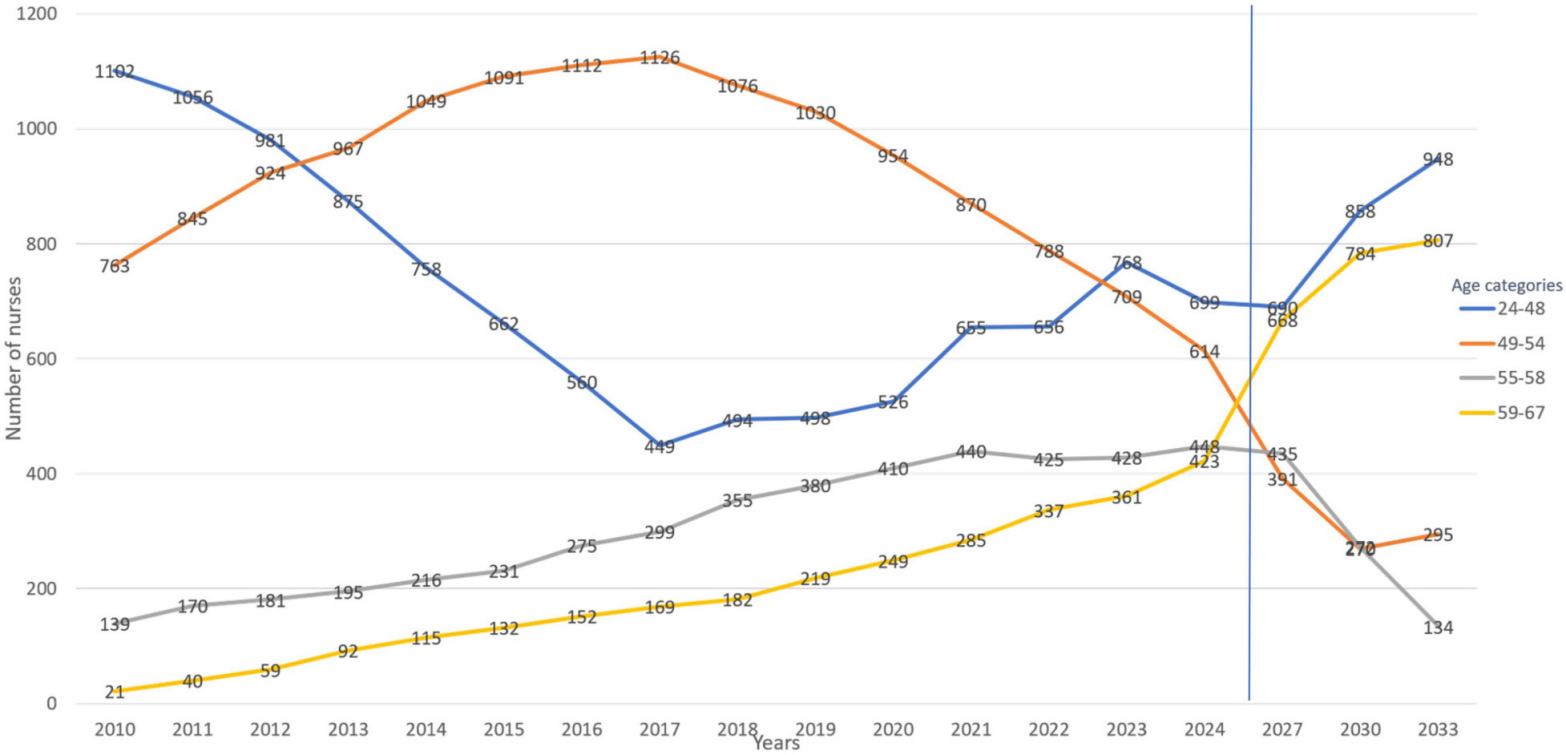
Aging nurse workforce trends through 2033. The vertical blue line highlights then end of the observation data (2024) and the beginning of the projection period.

The projection for 2033 is that the youngest age category (24–44) is expected to increase by 35.6%, from 699 to 948. At the same time, the oldest age category (59–67) will also increase by 91%, from 423 to 807. The intermediate categories are expected to decrease in the number of nurses, with the 45–54 age category showing a 52% decrease, from 614 to 295 and the 55–58 age category expected to decrease by 70%, from 448 to 134 nurses (Figure 1).

### 3.2 Work setting

**Figure 2:**
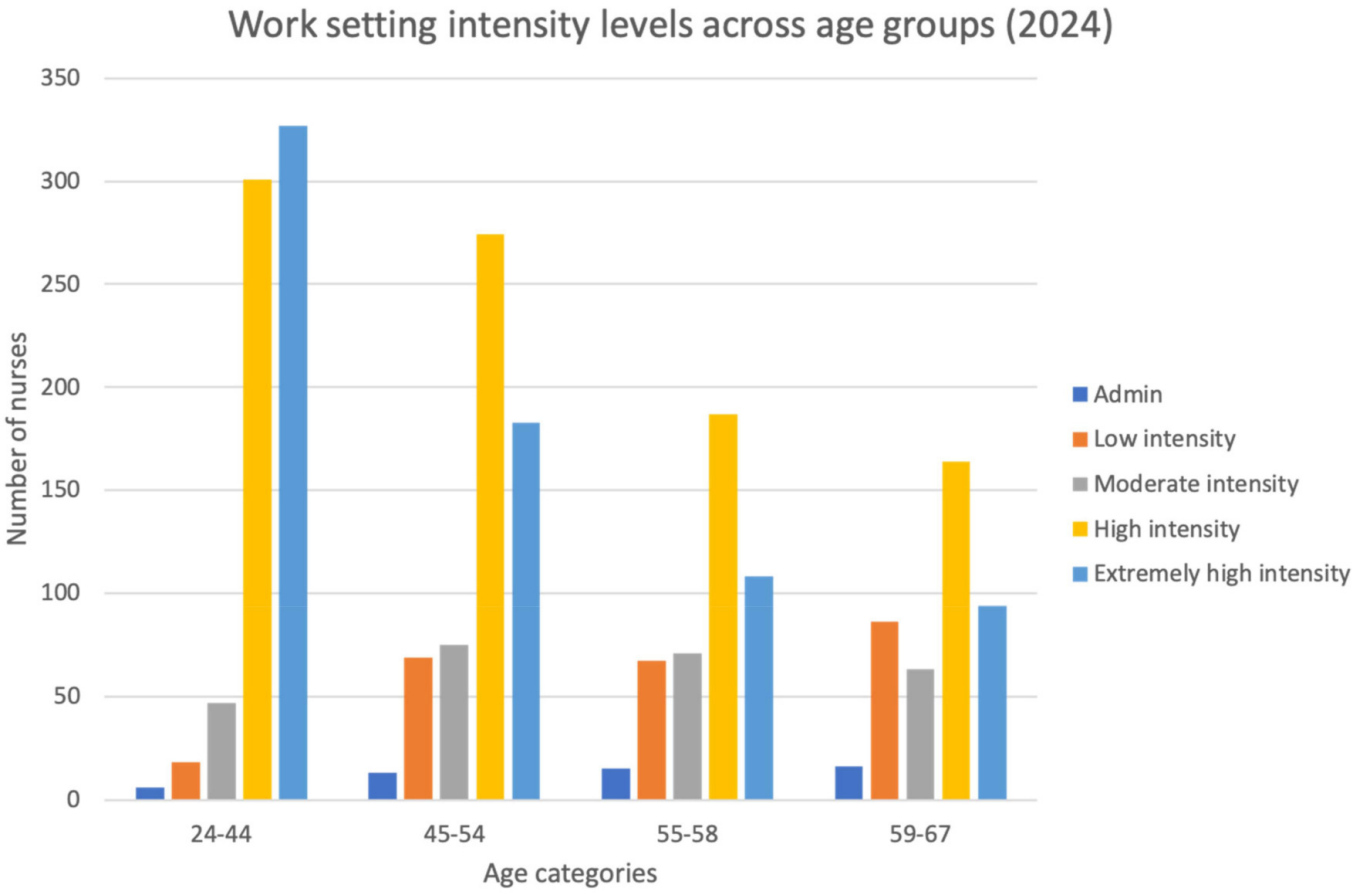
Current work setting by age category. The figure presents absolute counts.

In 2024, there were 290 nurses working in low intensity settings, 50 administrative and 240 in out-patient clinics. The youngest age category occupied 8.3% (*n* = 24) of these positions, the middle age categories both occupied 28.3% (*n* = 82) of the positions, and the highest age category occupied 35% (*n* = 102). Nearly 66% of nurses in the 55–58-year category (65.8%, 295 out of 448) and 61% of nurses over 59 years of age (258 out of 423) were working in high-intensity and extremely high intensity areas (Figure 2).

### 3.3 Medically documented health limitations

**Figure 3:**
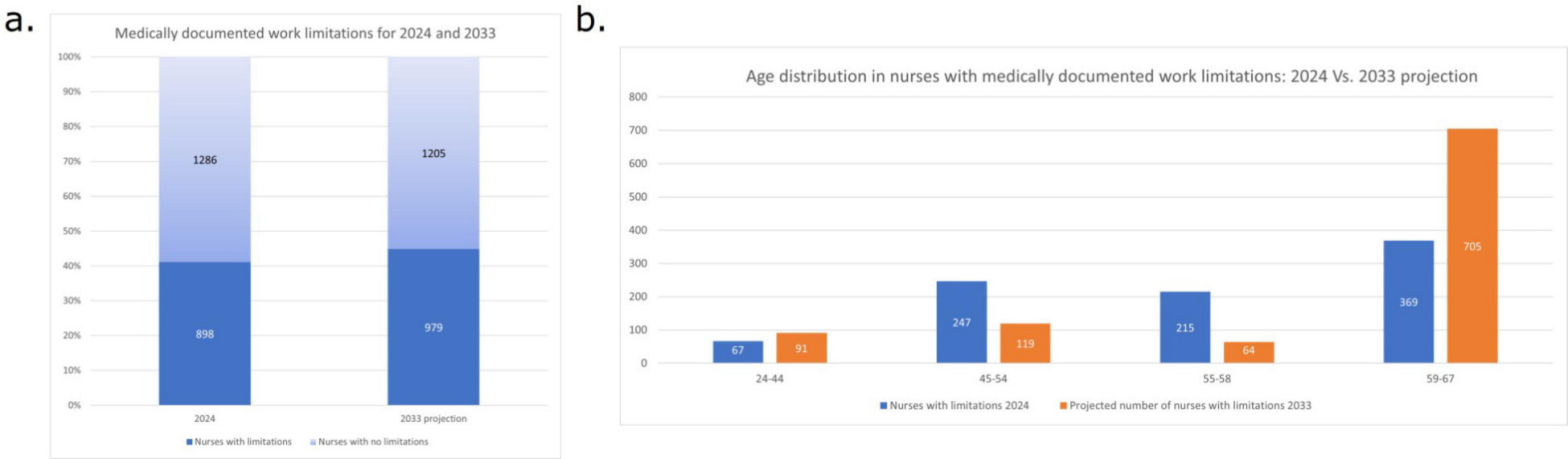
Medically documented work limitations in 2024 and projected in 2033 **(a)** with age distributions **(b)**.

In 2024, 9.5% (*n* = 67) of the youngest nurses had medically documented work limitations, while 40% of nurses aged 45–54 (*n* = 247) and 48% (*n* = 215) of nurses 55–58 years of age had health-related limitations. Out of 423 nurses over 59 years of age, 87% (n. 369) had medically documented work limitations. The projection for 2033 is that the total percentage of nurses with medically documented work limitations will increase from 41 to 44.8%. In the oldest age group, the percentage of nurses with limitations is projected to remain at 87%, but the raw number of nurses with limitations will increase from 369 to 705. Of these, 25.5% will be working in high and extremely high intensity areas (see Figure 3).

### 3.4 Health-related absenteeism

Based on data for the year 2024, the nursing staff had 35,998 days of health-related absenteeism, the full-time equivalent (FTE) of 160 nurses. Nurses in the oldest age category (59 to 67 years), approximately 19% of the nurse cohort in 2024, accounted for nearly one-third (29%) of missed workdays due to health reasons, the FTE of 47 nurses. Nurses in the older age category (>55 years, *N* = 871), who represent 40% of the total nurse cohort, accounted for 54% of missed days, equivalent to the FTE of 75 nurses.

## 4 Discussion

This study aimed to analyse the evolving demographic profile of the nursing workforce within a major Italian public hospital, with a specific focus on the aging nurse population. By examining current trends and projecting the future trajectory of this demographic, we sought to understand the potential implications of an increasingly older workforce on hospital operations and service delivery. We explored the distribution of older nurses across various clinical and non-clinical units to assess the alignment between age, role intensity, and workforce sustainability. Additionally, we investigated the prevalence of medically documented work limitations among older nurses and their impact on work attendance, as measured by health-related absenteeism.

### 4.1 Age

The age distribution of the nursing workforce has undergone a significant demographic shift over the 14-year period between 2010 and 2024, and future projections to 2033 suggest that these trends will continue, albeit with some notable changes. The data reveal a pronounced aging of the nursing workforce, which raises concerns for workforce sustainability, succession planning, and healthcare delivery.

In 2024, the mean age of nurses was 50.49 years with the majority (68%) aged 45 years or older. This represents a significant aging trend when compared to 2010, particularly among the most senior categories. The number of nurses aged 55–58 increased by an extraordinary 222.3%, and those aged 59–67 years increased by 1,914%. Some of this change may be due to the extended age of retirement as the magnitude of the increase suggests a long-term structural aging of the workforce rather than a temporary demographic spike. Alternatively, retirement waves from baby boomers, rather than pure structural aging, could be an additive driver of the relevant increase of the 59–67 group. Mid-career declines (45–58) could be related to voluntary exits due to stress, better awareness of work-life balance, or better opportunities elsewhere, offsetting younger nurse gains. Over the same period, the 36.5% decline in nurses aged 24–44 may be partly attributable to regional healthcare policies that periodically imposed hiring freezes or delays as cost-containment measures, thereby disrupting nurse recruitment and contributing to workforce aging by 2024.

It is worth considering that factors such as non-competitive salaries, less than ideal work environments and perceived lack of support from hospital administration may have exacerbated declines in the 24–44 year age group, independent of hiring freezes.

Looking ahead to 2033, projections show a mixed picture. On one hand, the anticipated 36% increase in younger nurses (from 699 to 948) is a positive development. However, this projected growth will be offset by a continued increase in the oldest age group (59–67 years), which is expected to nearly double (91% increase), while the mid-career cohorts (45–54 and 55–58 years) are projected to decline significantly (52% and 70%, respectively). These mid-career nurses are often critical in leadership, mentoring, and advanced clinical roles, and their decline could lead to skill and experience gaps within the system (8).

Collectively, these trends suggest that while some progress may be made in attracting younger nurses, the health system may face significant challenges from the concurrent loss of mid- and late-career expertise and an increasingly aging workforce. Strategic workforce planning will be essential to address these shifts, including enhanced recruitment and retention strategies for younger nurses, support for aging nurses, and policies that encourage knowledge transfer across generations. These solutions are strategic in nature, although a cultural inertia in public health systems like Italy's often impedes the planning and execution, particularly when “up front” investment is necessary.

Furthermore, the data highlight a need for proactive succession planning and the development of flexible employment models that can retain older, experienced nurses. Without such measures, the healthcare system may struggle with staffing shortages, reduced organizational memory, and increased stress on remaining staff (9).

Despite the projected influx of younger nurses, the broader trend toward an aging workforce presents significant risks. Addressing these challenges will require coordinated efforts across education, policy, and practice environments to ensure a resilient and sustainable nursing workforce.

### 4.2 Work setting intensity

The distribution of nurses across clinical settings in 2024 highlights an imbalance in the placement of the workforce related to age and work intensity. While over one-third of low intensity roles (35%, *n* = 102) were occupied by the oldest age group (59–67 years), a substantial proportion of older nurses, 65.8% of those aged 55–58 and 61% of those aged 59–67, were employed in high- and extremely high-intensity clinical settings.

This pattern suggests that a significant portion of the aging nursing workforce continues to be concentrated in roles that are physically and cognitively demanding. While this may reflect a strong commitment and clinical expertise among senior nurses, it raises concerns about workforce sustainability and occupational health. Further, the lack of reassignment options may also be an important factor in keeping older nurses in high-intensity environments despite risks.

As previously reported, older nurses are at increased risk of musculoskeletal injury, burnout, and fatigue, and continuing to staff them in high-intensity areas without adequate support may have long-term implications for workforce retention and may have negative consequences on both nurse and patient safety (10, 11).

These findings emphasize the need for age-sensitive workforce management strategies. As the workforce continues to age, health systems must consider transitioning older nurses into roles that align with their changing physical capabilities while still leveraging their expertise, particularly in areas of mentorship and peer and patient education. Currently the hospital under study has no formal policy supporting hybrid roles that combine clinical and administrative responsibilities, nor a structured process for redeploying older nurses to less physically demanding settings, partly due to the limited availability of such positions.

Additionally, the disproportionate number of older nurses in high-intensity settings further highlights an urgent need for succession planning. If a large segment of experienced nurses retires simultaneously, especially from demanding clinical areas, the system could face severe staffing shortages and knowledge gaps. Ensuring a balanced age distribution across clinical settings will be essential to maintain quality of care and operational resilience. Further, as demographic shifts continue, workforce policies must prioritize sustainable staffing models to optimize both nurse well-being and patient outcomes.

### 4.3 Medically documented work limitations

The findings from 2024 reveal a clear and concerning correlation between nurse age and the prevalence of medically documented work limitations. While less than 10% (*n* = 67) of the youngest nurses (24–44 years) had medically documented work limitations, which is to be expected, the proportion rose sharply with age, as shown in Figure 3. These patterns reflect the well-documented physiological and functional changes associated with aging, compounded by the physical and psychological demands of the nursing profession (12, 13). At the same time, it is important to consider that working for decades in a stressful environment, combined with shift work and long-term physical strain (prolonged standing, patient transfers, etc.) may appear as age-related but actually may also be explained by role-related and environmental factors.

This upward trend in medically documented work limitations is projected to continue. By 2033, the proportion of nurses with health-related limitations is expected to increase from 41% to 44.8%. Although the percentage in the oldest group is projected to remain constant at 87%, the absolute number of affected individuals will nearly double (from 369 to 705). This demographic shift represents a significant challenge for workforce planning and healthcare system resilience, particularly as 25.5% of these older nurses with limitations are projected to be working in high- and extremely high-intensity clinical environments.

The presence of medically documented work limitations among older nurses in demanding roles raises serious concerns regarding occupational health, and long-term system sustainability. Medically documented work limitations in older nurses in critical areas could also result in increased risk for patient safety, although this requires further investigation. High-intensity settings often require rapid physical responses, prolonged standing, and sustained cognitive alertness, capacities that can be compromised by chronic conditions, fatigue, and functional decline (14, 15). Continued reliance on an aging and physically limited workforce in these environments has been reported to increase the risk of burnout, injury, and diminished care quality (16).

The data again highlight a gap in workforce transition planning. Although age-related physical limitations are well documented, many older nurses remain in roles that may exceed their capacity. This likely reflects a shortage of flexible or adapted positions, insufficient health screening, and limited opportunities for redeployment into lower-intensity or supportive functions such as education, triage, or administration.

To address this growing mismatch, health systems must implement health-informed workforce planning. This involves proactive health surveillance, early identification of limitations, and the development of alternative roles that allow older nurses to contribute meaningfully while maintaining their health and safety (8, 17). Expanding mid- and late-career transition pathways, such as clinical educator roles, and hybrid positions, can also help retain institutional knowledge while reducing physical strain (18). As noted previously, there are currently no formal opportunities for hybrid nursing positions and this should be taken into consideration by decision-makers. Although the role of clinical nurse educator exists in very limited areas within the hospital, it requires a formally recognized job description, which currently is lacking.

### 4.4 Health-related absenteeism

The 2024 data on health-related absenteeism among nursing staff highlight a significant and growing challenge within the healthcare workforce, particularly related to aging. Over the course of the year, nurses collectively accounted for 35,998 days of absence due to health reasons, the full-time equivalent of 160 nurses. Strikingly, over half (54%) of these absences (19,588 days) were attributed to nurses ≥55 years of age, translating to the full-time equivalent of 87 nurses. These figures underscore the disproportionate rate of absenteeism by older nurses, reflecting the compounding impact of age-related health challenges previously identified in this workforce (19). As older nurses continue to represent a growing proportion of the overall staff, many of whom also have medically recognized work limitations, these findings emphasize the urgent need for workforce planning that accounts for both aging and occupational health demands related to working in a complex hospital environment.

The high rate of absenteeism among older nurses has broader implications for healthcare system performance, staffing stability, and quality of care. When chronic absenteeism is concentrated in one age group, it puts more strain on younger and mid-career nurses, increasing the risk of burnout and turnover (20). Additionally, the sudden or prolonged absence of experienced nurses can disrupt care continuity, training of new nurses, and leadership, key roles often filled by senior staff (21). This trend also suggests the potential cost of an aging workforce, including direct expenses like replacements and overtime, and indirect effects on team dynamics, productivity, and morale (17). If these trends continue unchecked, the system may face growing operational inefficiencies and increased dependency on temporary staffing solutions, which may not offer the same level of expertise or continuity (22).

To address this, healthcare organizations must adopt comprehensive age-sensitive strategies. These could include early health risk identification, more flexible scheduling, proactive wellness programs, and gradual transition plans for older nurses into less physically demanding roles (23). Encouraging part-time work, phased retirement, or hybrid administrative-clinical roles may help reduce the burden of absenteeism while still leveraging the experience of older nurses.

The 2024 health-related absenteeism data provide a critical indicator of workforce vulnerability tied to demographic change. As the nursing workforce continues to age, sustainable staffing will depend not only on recruiting younger nurses but also on managing the health and workload of senior staff more effectively. Future policies must integrate occupational health data into strategic workforce planning to ensure long-term system resilience and continuity of care.

### 4.5 Additional considerations

Two considerations should be noted regarding the reported data. First, the workforce projections to 2033 assume that current conditions remain largely unchanged, with stable hiring patterns and no major policy shifts affecting the nursing workforce, in particular the minimum age for retirement. Second, while nursing demographics may be broadly similar across a national health system, the findings are based on administrative data from a single large hospital in northern Italy, which may limit generalizability. Future studies could extend this analysis to a national scale.

## 5 Conclusions

This study highlights the urgent challenges of an aging nursing workforce which are not unique to Italy's NHS, but a global issue. In a large Italian hospital, data reveal a growing proportion of older nurses with medically documented work limitations working in high intensity areas, trends already testing healthcare systems in many countries worldwide with a potential impact on patient safety, quality of care, and workforce resilience.

Healthcare system decision-makers everywhere must act decisively, challenging deep-rooted organizational norms through innovative, evidence-based strategies, taking examples from best practice underway in a number of countries, like Switzerland, the Netherlands, Canada, and Australia. Flexible shift patterns, phased retirement, ergonomic redesigning, wellness programs, role redefinitions to leverage experience and intergenerational mentorship are all examples of innovative thinking that can redirect the nursing crisis (24, 25). Apart from retirement-age policy reforms, which fall within the scope of national regulation, the proposed strategies can be operationalized at the hospital level. Implementation efforts should prioritize collaborative governance models, including co-design with nurse labor representatives, to enhance feasibility, acceptability, and sustainability.

While financial constraints remain, possibly, the most significant barrier in the public hospital sector, failing to address the needs of an aging nursing workforce may ultimately result in higher long-term costs, through increased absenteeism, turnover, and compromised care quality. The “savings” of inaction could come at the expense of both staff well-being and system resilience. Ultimately, addressing the challenges of an aging nursing workforce will require proactive, coordinated and sustained efforts across education, policy, and clinical practice. Strategic planning must prioritize the development of a resilient, age-diverse nursing workforce that is equipped to meet the demands of an evolving healthcare landscape both today and in the years ahead. What is now needed is implementation research that involves countries with varying socio-economic realities in order to validate interventions and guide policy transformation.

## Data Availability

The original contributions presented in the study are included in the article/Supplementary Material, further inquiries can be directed to the corresponding author.
